# Intravesical High Dose BCG Tokyo and Low Dose BCG Tokyo with *GMCSF+IFN α * Induce Systemic Immunity in a Murine Orthotopic Bladder Cancer Model

**DOI:** 10.3390/biomedicines9121766

**Published:** 2021-11-25

**Authors:** Sin Mun Tham, Juwita N. Rahmat, Edmund Chiong, Qinghui Wu, Kesavan Esuvaranathan, Ratha Mahendran

**Affiliations:** 1 Department of Surgery, Yong Loo Lin School of Medicine, National University of Singapore, NUHS Tower Block, Level 8, 1E Kent Ridge Road, Singapore 119228, Singapore; surtsm@nus.edu.sg (S.M.T.); biejnr@nus.edu.sg (J.N.R.); surce@nus.edu.sg (E.C.); suresuva@nus.edu.sg (K.E.); 2 Department of Bioengineering, National University of Singapore, Singapore 119077, Singapore; 3 Department of Urology, National University Hospital, National University Health System, Singapore 119228, Singapore; qing_hui_wu@nuhs.edu.sg

**Keywords:** BCG Tokyo, combination of gene therapy and BCG, bladder cancer, antigen specific T cells, systemic immunity

## Abstract

This study evaluates a short therapy schedule for bladder cancer using BCG Tokyo. BCG Tokyo was evaluated in vitro using bone marrow derived dendritic cells, neutrophils, RAW macrophages and the murine bladder cancer cell line, MB49PSA, and compared to other BCG strains. BCG Tokyo > BCG TICE at inducing cytokine production. In vivo, high dose (1 × 10^7^ colony forming units (cfu)) and low dose (1 × 10^6^ cfu) BCG Tokyo with and without cytokine genes (*GMCSF + IFNα*) were evaluated in C57BL/6J mice (*n* = 12–16 per group) with orthotopically implanted MB49PSA cells. Mice were treated with four instillations of cytokine gene therapy and BCG therapy. Both high dose BCG alone and low dose BCG combined with cytokine gene therapy were similarly effective. In the second part the responsive groups, mice (*n* = 27) were monitored by urinary PSA analysis for a further 7 weeks after therapy cessation. More mice were cured at day 84 than at day 42 confirming activation of the immune system. Cured mice resisted the re-challenge with subcutaneous tumors unlike naïve, age matched mice. Antigen specific T cells recognizing BCG, HY and PSA were identified. Thus, fewer intravesical instillations, with high dose BCG Tokyo or low dose BCG Tokyo with *GMCSF + IFNα* gene therapy, can induce effective systemic immunity.

## 1. Introduction

High risk non-muscle invasive bladder cancer (NMIBC) is characterized by frequent recurrence. BCG immunotherapy reduces the incidence of recurrence and progression [1] when given post tumor removal. BCG was originally derived by attenuation of Mycobacterium bovis and was distributed to several countries where prolonged propagation led to the development of the different strains available today. Today’s strains have some genetic losses, which correlate with different immunostimulatory capacity [2], cytokine production and cytotoxicity on human bladder cancer cell lines and immune cells [3,4]. BCG Connaught, Glaxo, Moreau, RIVM, Pasteur, Tice and Tokyo have been used clinically but few comparison trials have been satisfactorily completed [5]. BCG Tokyo was reported to be as efficacious as BCG Connaught in a clinical trial that was not completed due to the worldwide shortage of BCG Connaught [6]. BCG induces cytokine production (IL2, IL6, IL8, IL10, IFNγ, TNFα) during therapy, which can be measured in urine and serum [7,8], and these cytokines are believed to be mediators of the immune response to BCG as poor responders have lower cytokine production [8]. It is believed that the cytokines both recruit and activate immune cells [9]. In an in vitro study, blocking cytokine production had an impact on macrophage mediated cytotoxicity [10,11]. Given their role in immune modulation, cytokines have been combined with BCG therapy, the most common being recombinant IFNα [12]. IFNα has both direct cytotoxic effects on cancer cells and aids dendritic cell maturation, which is important for T cell activation [13].

Bladder tissue analysis in patients receiving BCG immunotherapy have shown a correlation between exhaustion of T cells and poor response to therapy [14,15]. Following intravesical therapy, there were shown to be increased T cells to BCG antigens [16]; however, tumor antigen specific T cells have not yet been confirmed. Orthotopic rodent and murine bladder tumors treated with BCG (Pasteur, Connaught and Tice) were shown to recruit both CD4 [17,18] and CD8 [18,19] T cells that recognized ‘tumor antigens’ overexpressed in the tumor cell line [18] and BCG antigens [18,19,20]. BCG Connaught was better than BCG Tice at inducing T cell recruitment in the murine bladder [19].

For high risk patients, the European Association of Urology (EAU) recommends six instillations of BCG followed by three weekly instillations at the third and sixth months and every 6 months (maintenance therapy) thereafter for 3 years [21]. BCG therapy is associated with adverse effects, namely, severe cystitis and contracted bladders. Thus, lower doses of BCG colony forming units (cfu) have been evaluated in clinical therapy. Martinez-Pineiro reported no difference in efficacy between a one-third dose and full dose of BCG Connaught [22] but the one-third dose of BCG TICE was inferior to the full dose [23]. Thus, BCG strain and dose impact clinical outcomes [24].

In mice, persistent Mycobacterial antigen stimulation results in dampening of antigen specific T cell numbers and function [25]. Repeated intravesical instillations are comparable to repeated antigen stimulation. Aging is associated with immune impairment in innate and adaptive immune cells and as patients with bladder cancer are older, multiple instillations of BCG may result in an inferior response [26]. In patients with previous BCG exposure, increased lymphoproliferative responses to BCG proteins were observed after four instillations [27]. In animal studies, shortened intravesical therapy schedules are efficacious in curing bladder tumors [28]. Mathematical modelling indicated that increased BCG cfu can be beneficial and that reducing the number of instillations may be efficacious [29]. Shah et al. demonstrated that a higher BCG Tice cfu (4 × 10^7^ cfu/0.1 mL) reduced tumor growth in murine bladders after three instillations [30]. We hypothesize that reducing the number of instillations when using higher doses of BCG should reduce associated side-effects and dampening of the immune response.

BCG Tokyo and Tice have been used in clinical practice in Singapore as replacement for BCG Connaught, which ceased production in 2012. In this study, we chose to evaluate the immunostimulatory capacity of the three BCG strains: Connaught, Tokyo, and Tice in vitro before evaluating different doses of BCG Tokyo over four instillations in a murine orthotopic model of bladder cancer with and without *GMCSF + IFNα* gene therapy. The rationale for using cytokine gene therapy in combination with BCG was to determine if it could enhance the response to BCG Tokyo. GMCSF activates macrophages and promotes dendritic cell maturation, thus *GMCSF + IFNα* expression would lead to recruitment of innate immune cells and their maturation. We set out to evaluate T cell responses post-BCG therapy in response to BCG Tokyo. The therapy schedule consisted of alternating cytokine gene therapy and BCG instillations [31].

## 2. Materials and Methods

### 2.1. Plasmids and BCG

In vivo grade pBud-Gmcsf + Ifnα (plasmid containing murine cytokine genes *Gmcsf* and *Ifnα*) with low endotoxin (<5 EU/mg) was prepared by Aldevron, RRID:SCR_011017 (Fargo, North Dakota). Lyophilized BCG strains, BCG Connaught (ImmunCyst^®^, Sanofi S.A., Paris, France), BCG Tice (OncoTICE^®^, Merck Sharp & Dohme, NSW, Australia) and BCG Tokyo 172 (Immunobladder^®^ Intravesical, Japan BCG Laboratory, Tokyo, Japan) were reconstituted immediately before use. Clumped BCG was centrifuged at 700 rpm for 2 min and bacteria in the suspension phase was harvested. BCG cfu were determined by plating the BCG on Middlebrook 7H10 agar plates supplemented with 10% ADC supplement, 0.05% Tween-80 and 0.5% glycerol.

### 2.2. Effect of BCG Strains on Immune Cells In Vitro

Bone marrow derived cells (BMDCs) were extracted from the femurs and tibias of 6–8 weeks old C57BL/6 mice (IMSR Cat numberJAX:000664, RRID: IMSR_JAX:000664). Dendritic cells (DCs) were generated by growing BMDCs in RPMI complete media (with 10% FBS, 2 mmol/L L-glutamine and 0.05 mg/mL Penicillin-Streptomycin) supplemented with 1% HEPES, 1% MEM, 50 µM β-mercaptoethanol, 0.1% sodium pyruvate and 40 ng/mL murine GM-CSF for 9 days as previously described [32]. The growth media is changed every 3 days. Neutrophils were isolated from BMDCs using the EasySep™ neutrophil enrichment kit (StemCell Technologies, Vancouver, Canada). Neutrophils and DC (5 × 10^5^) were treated with BCG at a ratio (multiplicity of infection (MOI)) of 1 cell: 5 BCG for all BCG strains (Connaught, Tice and Tokyo) for 2 h. BCG containing media were removed, remaining BCG killed with media containing 200 μg/mL gentamicin for 2 h and further incubated with fresh media with 20 ng/mL GM-CSF for 16 h. For neutrophil-DC co-culture experiments, DC (2.5 × 10^5^) were added to BCG-treated neutrophils after removal of BCG that was not internalized by neutrophils. Concentration of cells were kept at 5 × 10^5^ cells/mL. The supernatants were collected and assayed for cytokines (TNFα, IL-10 and IL-2) using ELISA.

### 2.3. In Vitro Stimulation of Tumor Cells and Macrophages with BCG Tokyo and IFNα

MB49PSA murine bladder cancer cell-line (grown in complete DMEM) and RAW264.7 murine macrophages cell-line (ATCC catalogue number TIB-71, RRID: CVCL_0493) (grown in complete DMEM supplemented with 1 mM sodium pyruvate) were plated at 2.5 × 10^5^ cells/well one day before treatment. Cells were exposed to BCG singly or in combination with 1 ng/mL recombinant IFNα (Biolegend, San Diego, CA, USA). BCG doses were set at MOI 1:1 or 1:5 for RAW264.7 and MOI 1:4 or 1:40 for MB49PSA to evaluate the effects of low and high dose BCG. After being exposed to BCG for 24 or 48 h, IL-6, TNFα and IL-10 were measured in the supernatant by ELISA (Thermo Fisher Scientific, Waltham, MA, USA) and cell proliferation was measured by enumerating cells after trypan blue staining. RAW264.7 were treated with BCG for 2 h as well. BCG containing media were removed, and the remaining BCG was killed as described above, and cells were further incubated with fresh media for 24 h.

### 2.4. Orthotopic Tumor Model

All animal work adhered to the Institutional Animal Care and Use Committee (IACUC) guidelines on animal use and handling at the National University of Singapore (084/12(A4)16). Five to six-week-old female C57BL/6J mice were orthotopically implanted with MB49PSA (human prostate-specific antigen secreting MB49 cells) [33] using poly-L-lysine [34]. Seven days later mice were randomly assigned to treatment groups (*n* = 10). Urinary PSA levels were measured using the free PSA (Human) ELISA kit (Abnova, Taipei City, Taiwan) and normalized to creatinine measured with the Quantichrom™ Creatinine Assay Kit (BioAssay Systems, Hayward, CA, USA) as previously described [33]. At termination, mice bladders were snap frozen and RNA was extracted by homogenizing the tissue in Trizol^®^ (Thermo Fisher Scientific). PSA gene expression in the bladders was quantified via real-time PCR.

### 2.5. Treatment Groups

Therapy was instilled into the bladder, in a volume of 0.1 mL using the sheath of a 24G i.v. catheter. The sheaths were left in place with a stopper for 2 h. As a control for gene therapy, the empty vector pBudCE4.1 was used for transfection and control mice received saline to control for BCG instillations. There were six treatment groups: control (empty vector pBudCE4.1 and saline); cytokine gene therapy (pBud-Gmcsf + Ifnα and saline); high dose BCG therapy (pBudCE4.1 and 10^7^ cfu BCG); low dose BCG therapy (pBudCE4.1 and 10^6^ cfu BCG); combined-high dose (pBud-Gmcsf + Ifnα and 10^7^ cfu BCG) and combined-low dose (pBud-Gmcsf + Ifnα and 10^6^ cfu BCG). All mice received 4 instillations of gene therapy and 4 instillations of BCG therapy. The experiment was performed twice and the data from both sets pooled. A liposome-based transfection system was used for gene delivery as previously described [35]. The treatment plan for the short-term experiment is to treat mice for 4 weeks and then cull them a week after the last instillation. In the re-challenge experiment, mice were monitored for a further 8 weeks by measuring urinary PSA. Mice were re-challenged with MB49-PSA cells implanted subcutaneously on the right flank and tumor growth was monitored every 3 or 4 days with calipers for 2 weeks. The tumor volume was calculated using the formula v = (ab^2^)/2 where ‘a’ is the longest dimension and ‘b’ the perpendicular width, both in mm. Immune populations in the spleens were analyzed via flow cytometry at the endpoint. This section was performed in 2 parts, first with 12 mice in each treatment group and 5 control age matched mice and in the second part with 15 mice in each treatment group and 5 control age matched mice.

### 2.6. Flow Cytometry

Spleens were digested with 2 mg/mL collagenase D (Merck, Kenilworth, NJ, USA) at 37 °C for 30 min and passed through a 70 µM cell strainer (Becton Dickinson, Franklin Lakes, NJ, USA). Red blood cells were lysed with ACK buffer (0.15 M NH_4_Cl, 10 mM KHCO_3_, 0.1 mM EDTA) for 5 min at room temperature. Immune cells were stained with antibodies to T cells, neutrophils, macrophages and NK cells for 30 min on ice in staining buffer (phosphate-buffered saline with 0.01% sodium azide and 1% bovine serum albumin) and fixed in fixation buffer (staining buffer with 1% formaldehyde). The antibodies used for staining were from BD Biosciences: FITC-conjugated anti-CD3 (Cat number 555274, RRID:AB_395698), PE-conjugated anti-CD4 (Cat number 553653, RRID:AB_394973); Thermo Fischer Scientific: APC-conjugated anti-CD8a (Cat number 17-0081-82, RRID:AB_469335), PerCP-Cy5.5-conjugated anti-Ly-6G (Cat- number 45-5931-80, RRID:AB_906247) and BioLegend: PerCP-Cy5.5-conjugated anti-CD278 (Cat number 313517, RRID:AB_10639735), PE-Cy7-conjugated anti-F4/80 (Cat number 123113, RRID:AB_893490) and AF647-conjugated anti-NK-1.1 (Cat number 108720, RRID:AB_2132713).

LIVE/DEAD fixable cell stain (Thermo Fisher Scientific) was used to gate live lymphocytes for the pentamer staining. BCG-, PSA- and HY-specific T cells were separately incubated with the R-PE labelled Pro5 murine H-2Db/GAPINSATAM (BCG)/HCIRNKSVI (PSA)/KCSRNRQYL (HY antigen) pentamers (Proimmune, Oxford, England) in staining buffer at room temperature for 10 min according to the manufacturer’s instructions. After one wash, cells were stained with secondary antibodies for 30 min on ice. The antibodies used were from Thermo Fischer Scientific: PerCP-Cy5.5-conjugated anti-CD3e (Cat number 45-0031-82, RRID:AB_1107000), APC-conjugated anti-CD8a (Cat# 17-0081-82, RRID:AB_469335), FITC-conjugated anti-NK-1.1 (Cat number 11-5941-81, RRID:AB_465317), FITC-conjugated anti-CD11b (Cat number 11-0112-81, RRID:AB_464934), FITC-conjugated anti-F4/80 (Cat number 11-4801-82, RRID:AB_2637191), FITC-conjugated anti-CD4 (Cat number 11-0041-82, RRID:AB_464892) and FITC-conjugated anti-CD45R/B220 was from BioLegend (Cat number 103206, RRID:AB_312991). In all samples, anti-mouse CD16/CD32 antibodies (Thermo Fisher Scientific Cat number 14-0161-82, RRID:AB_467133) were added before staining to block non-specific binding. To ensure accurate detection of these antigen specific T cells, live cells were first gated using the forward-scatter and side-scatter gates. To reduce non-specific binding of pentamers to other cells, NK-1.1, CD11b, F4/80, CD4, CD45R/B220 and dead cells were excluded by negatively gating on stained cells and plotting CD3^+^CD8^+^pentamer^+^ cells. Stained samples were analyzed on the BD FACSCanto™ flow cytometry system. Acquisition and analysis of samples was performed using the BD FACSDiva Software (RRID:SCR_001456) version 7. The proper compensation during data collection was set using BD CompBead. Tumor bearing mice and normal mice were evaluated to produce a mean + 3 SD value above which pentamers were deemed positive for tumor antigens PSA and HY in the pentamer analysis. For the BCG antigens, all tumor bearing mice not treated with BCG were used to produce the mean + 3 SD value. For the re-challenge study, control mice were analyzed to produce the mean +3 SD cut off value above which pentamers were deemed positive for antigens.

### 2.7. Real-time PCR Analysis and RT-PCR

Mice were terminated at day 42 for the short-term study. Gene expression profiling was performed on 3 bladder RNA samples per treatment group (low dose, high dose and combined therapy) using Affymetrix Clariom™ D Pico Assay (mouse) arrays (Thermo Fisher Scientific). Following the manufacturer’s protocol, 10 ng RNA was reverse transcribed with the poly-A tail initiation at a low cycle PCR followed by linear amplification using T7 in vitro transcription to produce cRNA. The cRNA was converted to cDNA, fragmented, labelled and hybridized to Clariom™ D Pico Assay (mouse) arrays for 16 h at 45 °C with rotation at 60 rpm. Arrays were then washed, stained and scanned using the Affymetrix 3000 7G scanner. Data analysis was performed using the Affymetrix Transcriptome Analysis Console Software (RRID:SCR_018718) version 3.1 to identify differentially expressed genes. Real-time PCR reactions were performed for a select group of genes from the array to validate the array data. The pre-formulated TaqMan™ gene expression assays (Thermo Fisher Scientific) for real-time PCR was performed for *PSA* Hs00426859_g1; *Arg1* Mm00475988_m1; *Ccl24* Mm00444701_m1; *CD163* Mm00474091_m1; *CD274* Mm03048248_m1; *Cd276* Mm01179456_m1; *CD86* Mm00444543_m1; *Cldn8* Mm00516972_s1; *Il10* Mm00439614_m1; *Il24* Mm00474102_m1; *Impdh2* Mm00496156_m1; *Krt5* Mm00503549_m1; *Mmp10* Mm01168399_m1; *Mmp13* Mm00439491_m1; *Mmp7* Mm00487724_m1; *Mst1* Mm01229834_m1; *Nos2* Mm00440502_m1; *Odc1* Mm02019269_g1; Slc2a1 Mm01192270_m1; Tgfb1 Mm01178820_m1; *Timp1* Mm01341361_m1. Samples were assayed in triplicate and normalized with *18S* (Hs99999901 s1).

### 2.8. Statistical Analysis

Statistical analyses were performed with SPSS (RRID:SCR_002865) version 24. In the in vitro experiments, one-way ANOVA (Dunnett’s Test) was used to compare experimental groups against the control. To analyze variance in gene expression between the animals, one-way ANOVA (Bonferroni) was used. To perform further analysis of gene expression between cured mice and tumor bearing mice, independent samples *T*-test was used. A *p*-value, *p* < 0.05 was deemed to be statistically significant. Survival and cure rates were expressed as a percentage.

## 3. Results

### 3.1. Comparison of Cytokine Induction by the Three BCG Strains

**Figure 1:**
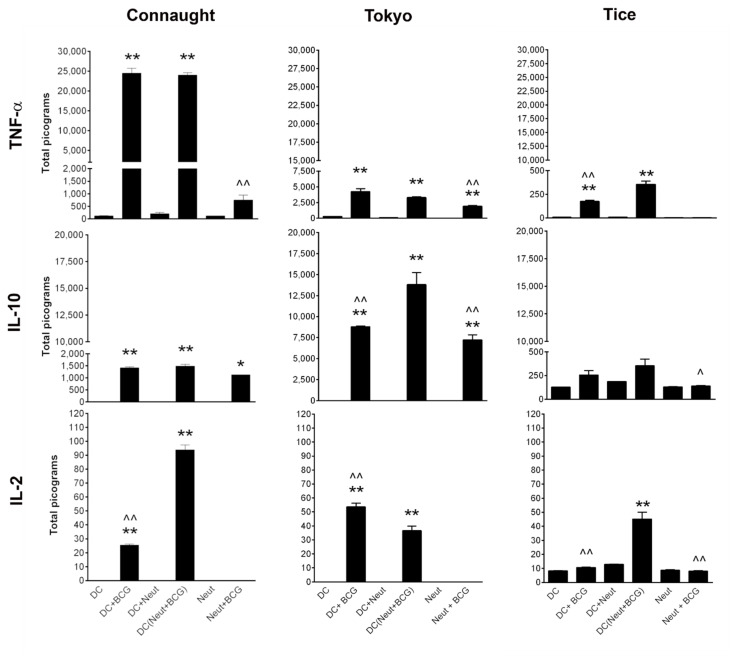
In vitro cytokines produced by murine bone marrow-derived dendritic cells (DC) and neutrophils (Neut) stimulated with BCG Connaught, BCG Tokyo and BCG Tice strains at MOI of 1:5. Immune cells were exposed to BCG for 2 h and then removed. TNFα, IL-10, and IL-2 production were measured in the supernatant 16 h later. In DC (neut + BCG), neutrophils were first exposed to BCG for 2 h and DC was stimulated with BCG activated neutrophils. One-way ANOVA with a Bonferroni post-hoc analysis was used for comparison among multiple groups. * denotes significance compared to respective control and ^ denotes significance compared to DC (Neut + BCG) group. *, ^ *p* < 0.05, **, ^^ *p* < 0.005.

A comparison was made of the three strains on murine DC and neutrophils at a multiplicity of infection (MOI) of one cell to five bacteria (1:5) [36]. Immune cells were exposed to BCG for 2 h, followed with BCG being removed and cytokine production (TNFα, IL-10 and IL-2) was measured 16 h later, Figure 1. TNFα is an inflammatory cytokine, IL10 is anti-inflammatory and IL2 is a cytokine that stimulates lymphocyte. Thus, evaluating these three cytokines provides a view of the immunostimulatory capacity of the DC and neutrophils stimulated with BCG.

DC and neutrophils were stimulated with BCG and DC were also stimulated with BCG activated neutrophils. BCG Connaught was a strong stimulator of DC. DC stimulated with BCG Connaught activated neutrophils had increased IL-2 production. DC treated with BCG Tokyo produced 6-fold less TNFα, 10-fold more IL-10 and 2-fold more IL-2 compared to BCG Connaught. BCG Tokyo was also a stronger activator of neutrophil IL-10 production than BCG Connaught. In contrast, BCG Tice induced less cytokines, Figure 1. Compared to BCG TICE, BCG Tokyo was more similar in its immunostimulatory profile to BCG Connaught.

### 3.2. Dose Response of BCG Tokyo on Cytokine Production and Cell Proliferation

The impact of different doses of BCG Tokyo was examined on the murine bladder cancer cell line MB49PSA [33] and RAW264.7 macrophages in terms of cytokine production and cell proliferation. IL6 and TNF α are inflammatory cytokines that also have direct cytotoxic effects on cancer cell lines and are able to recruit immune cells. IL10 in contrast has a role in downregulating the inflammatory response and promoting a TH2 type response. Thus, evaluating these three cytokines provides a picture of the immune stimulatory capacity of the three strains.

**Figure 2:**
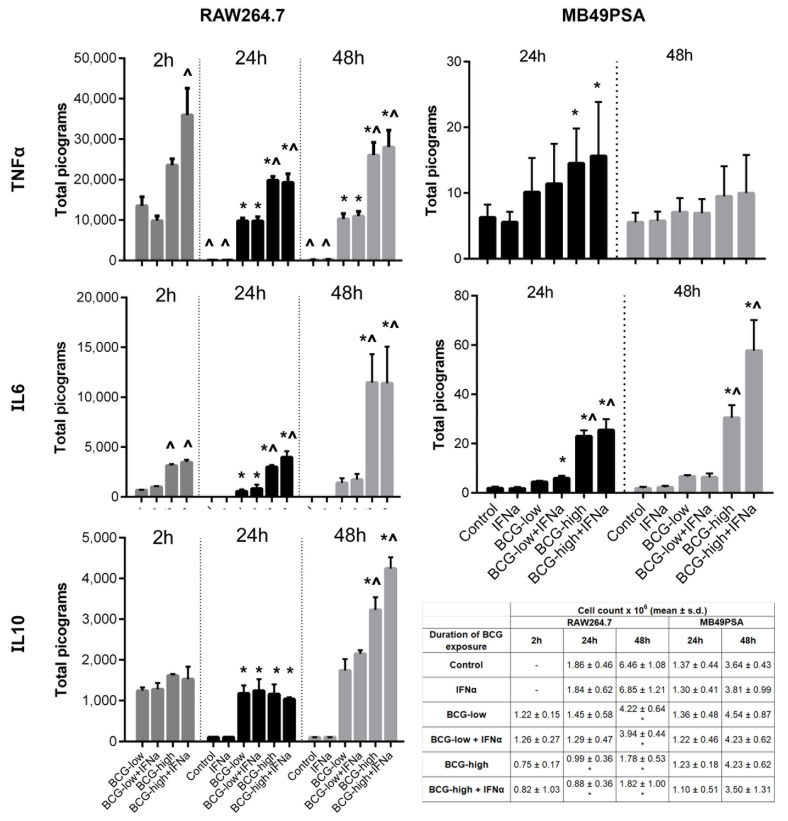
Cytokine production and cell proliferation in RAW264.7 macrophages and MB49PSA cells stimulated with different doses of BCG Tokyo singly or in combination with recombinant IFNα. BCG-low refers to low dose BCG, which is set at MOI of 1:1 for RAW264.7 and 1:4 for MB49PSA. BCG-high refers to high dose BCG (MOI 1:5 for RAW264.7 and 1:40 for MB49PSA). After being exposed to BCG for 24 or 48 h, TNFα, IL-6 and IL-10 production were measured in the supernatant. The effect of a short 2 h exposure to BCG was also examined on RAW264.7. * *p* < 0.05 one-way ANOVA (Dunnett’s *T* test, against Control) ^ *p* < 0.05 one-way ANOVA (Bonferroni, against BCG-low). Cell proliferation was measured after exposure to BCG for 24 and 48 h for MB49PSA cells and after exposure to BCG for 2 h, 24 and 48 h for RAW264.7 macrophages. For the cell proliferation data * *p* < 0.05 one-way ANOVA (Dunnett’s *T* test, against Control).

In clinical practice, BCG has often been combined with IFNα with good effect [37]. Therefore, BCG Tokyo combined with IFNα was also studied. BCG doses were set at MOI 1:1 (low dose) or 1:5 (high dose) for RAW264.7 and MOI 1:4 (low dose) or 1:40 (high dose) for MB49PSA. There was a low level of increased TNFα and IL-6 secretion from MB49PSA cells after 24 h of exposure to high dose BCG with and without IFNα. Interestingly, only IL-6 secretion continued to increase after 48 h of continuous exposure, Figure 2. In RAW254.7 macrophages, TNFα production was higher when high dose BCG was combined with IFNα at 2 h. Otherwise, combined therapy did not result in a higher production of IL-6, IL-10 and TNFα with time. Overall, all cytokines increased with longer exposure to BCG and with higher dose. Both low and high dose BCG reduced cell proliferation of RAW254.7 macrophages (Figure 2). Even a short 2-h exposure to BCG was sufficient to reduce RAW264.7 macrophage survival and the addition of IFNα did not enhance this effect.

### 3.3. Impact of High and Low Dose BCG Tokyo Alone or Combined with Cytokine Gene Therapy on Orthotopic Bladder Tumors in Mice

**Figure 3:**
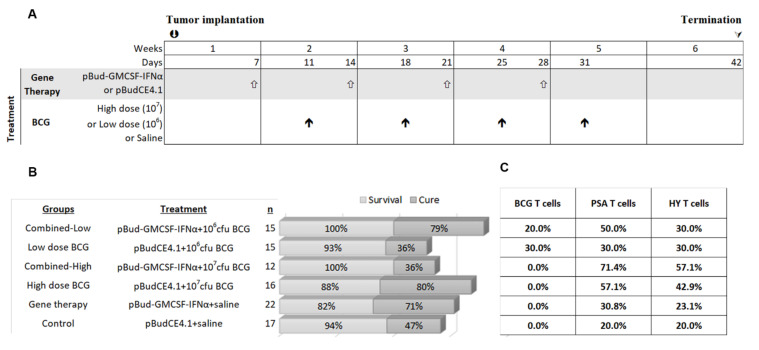
The schedule and outcome of 4 instillations of gene therapy and high and low dose BCG. (**A**) The experimental plan and schedule of instillations are shown as well as experimental groups. (**B**) The outcome of the short-term study, in terms of cure rate and survival, where the cure rate was determined by real-time PCR for the presence of the marker gene PSA. (**C**) The percentage of mice with antigen specific T cells recognizing BCG, PSA and HY in each therapy group are shown.

High dose BCG Tokyo (1 × 10^7^ cfu/0.1 mL), low dose BCG Tokyo (1 × 10^6^ cfu/0.1 mL) and these doses combined with pBud-GMCSF-IFNα gene therapy were evaluated using the therapeutic schedule shown in Figure 3. There were six groups in total: control, high dose BCG; low dose BCG; cytokine gene therapy; combined-high dose BCG (cytokine gene therapy combined with high dose BCG) and combined-low dose BCG. Therapy commenced seven days after orthotopic tumor implantation in mice. Gene therapy was administered three days prior to BCG as this was previously shown to improve response to BCG [31]. Weekly urinary PSA was monitored until day 42. The cure rate, Figure 3, for combined-low dose BCG was 79%, while it was 36% for low dose BCG alone. For high dose BCG, the cure rate was 80% but for the combined-high dose BCG, the cure rate was only 36%. The spontaneous cure rate in control mice was 47%. Thus, high dose BCG alone or combined-low dose BCG achieved similarly high cure rates of 30% more than the spontaneous cure rate, Figure 3.

Immune activation was examined by analyzing spleen for the presence of antigen specific T cells. Pentamers recognizing the HY antigen (expressed on MB49 cells as they were originally generated in male mice) [38], PSA (MB49 cells were transfected with human PSA gene) [33] and BCG antigens [20] were used for flow analysis. The percentage of mice with T cells recognizing PSA and HY antigens was about 20% in the control group. In the combined-high dose BCG treated group, 71% had PSA positive T cells and 57% HY positive T cells but the cure rate was poor in this group (Figure 3C). In the high dose BCG, 57% of mice were positive for PSA and 43% for HY antigen, and in the combined-low dose BCG group 50% were positive for PSA and 30% positive for HY (Figure 3C). Despite the presence of these T cells, tumors persisted in some mice while mice without antigen positive T cells were cured. This indicates that the tumor cells were immunogenic but local immune inhibitory measures could be blocking tumor eradication, even if tumors were cleared by other immune cell types such as by NK activation. The percentage of mice with T cells recognizing BCG antigens appeared to be low.

### 3.4. Gene Expression in the Local Environment in Response to Therapy

To determine if there were differential gene expression changes associated with therapy, mice bladders were harvested and the RNA was used to probe an Affymetrix array. We focused on gene expression changes in the coding genes. When comparing mice treated with high dose BCG and combined-low dose BCG, there were fairly similar gene expression changes with fewer genes being upregulated and more being downregulated (*p* < 0.05), as shown in Figure S1. A comparison of cured and tumor bearing mice showed greater upregulation of some genes (Figure S1). Selected genes from the array, as shown in Table S1, were validated in all mice. The genes selected encompassed those related to proliferation; epithelial mesenchymal transition, wound healing and immune activation. PSA mRNA levels were used to segregate cured and tumor bearing mice. The *IL24* gene was downregulated across all three treatment arms and was expressed at very low levels in cured mice (*p* < 0.05 one-way ANOVA Bonferroni), as shown in Table S2. *MMP10*, *7*, *13* and *TIMP1* were reduced in cured mice compared to tumor bearing mice, while *Krt5* and *Cldn8* levels increased in cured mice. *CCL24* was increased in treated tumor bearing mice and was reduced in cured mice. *Arg1* and *Odc1* was reduced in cured mice (*p* < 0.05 for low dose BCG cured versus tumor bearing mice). There was no change in *Nos2* levels in all treatment groups.

The gene expression data showed clear differences between cured and tumor bearing mice but no significant differences between tumor bearing high dose BCG and combined-low dose BCG treated mice. In tumor bearing mice *CCL24*, *CD163* and *MMP7* were upregulated after therapy and *IL24* and *Odc1* were downregulated (*p* > 0.05).

### 3.5. Re-Challenge of Cured Mice

**Figure 4:**
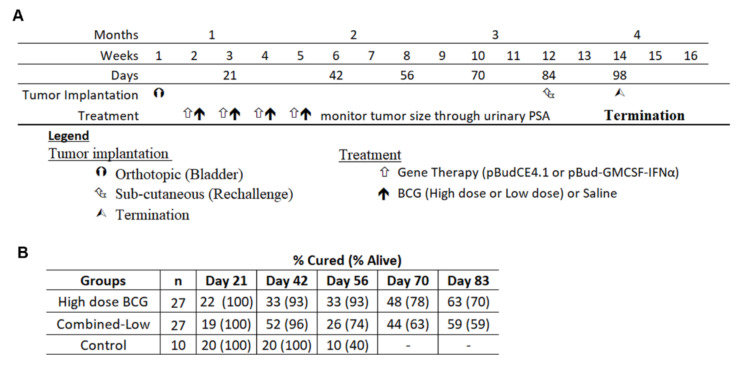
The experimental plan for the long-term experiment and monitoring of tumor growth. (**A**) The plan of therapy and long-term monitoring are shown and dates when urine was collected for analysis of PSA. (**B**) The cured status was determined by measuring urinary PSA and normalizing to creatinine levels.

For the re-challenge study, to determine if systemic responses were generated, mice were treated with high dose BCG and combined-low dose BCG therapy on the same schedule and monitored for 84 days. The combined data of two independent experiments is shown in Figure 4A. Urinary PSA was measured weekly in these mice to chart tumor growth. Despite the cessation of therapy on day 31, PSA levels continued to change, indicative of on-going anti-tumor activities and of successful immune activation (Figure 4B). Some mice cured at day 31 exhibited tumor recurrence at a later time point while mice still bearing tumors when therapy ceased were later cured, as shown in Figure 4B. The single control mouse that was alive was culled at day 64 and found to be tumor free. By day 83, 19/27 mice were alive in the high dose group. Out of these 19 mice, 17 were cured (63% overall) and the remaining two had very small tumors. While 16 mice of the combined-low dose therapy group were alive, the cure rate was 15/27 (56% overall) and one mouse had a small tumor. Thus, high dose BCG alone or combined-low dose BCG achieved respective cure rates of 53% and 46% more than the spontaneous cure rate (10%).

**Figure 5:**
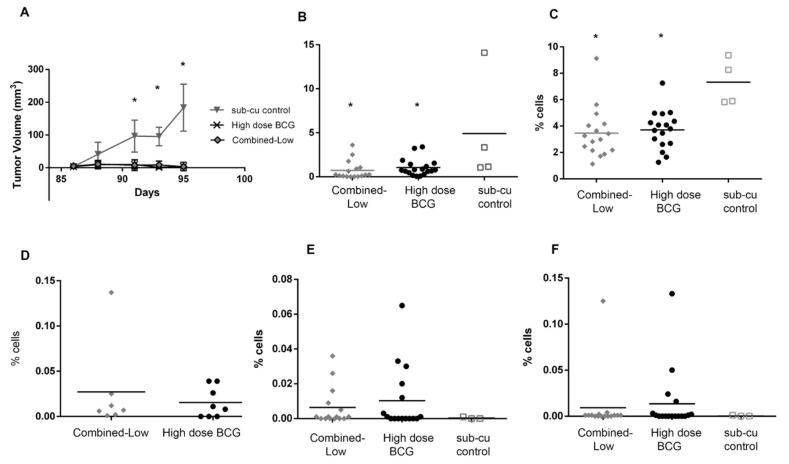
Response to re-challenge with MB49PSA tumor cells. (**A**) Response to subcutaneous tumors in cured and aged matched naïve mice was monitored for 10 days and expressed as tumor volume with time (* *p* < 0.05 one-way ANOVA (Bonferroni, against sub-cu control). Splenocytes were isolated from mice in all three groups and analyzed by flow cytometry for (**B**) CD4^+^ICOS^+^ T cells and (**C**) NK cells (* *p* < 0.05 one-way ANOVA (Bonferroni, against sub-cu control).). Antigen specific T cells recognizing (**D**) BCG, (**E**) PSA and (**F**) HY.

All the mice alive at day 84 were re-challenged with subcutaneous implantation of MB49PSA cells along with age matched naïve healthy mice to determine the presence of systemic immunity. While the tumors grew in all age matched mice, as shown in Figure 5A, the cured mice resisted tumor growth and remained tumor free. The mice from both groups with small tumors in the bladder at the point of re-challenge also resisted tumor growth but their small bladder tumors remained at day 98.

The immune populations in the spleen were analyzed and both the treated groups had lower CD4^+^ICOS^+^ T cell and NK cells in their spleens than tumor bearing age matched mice, as shown in Figure 5B,C. The reduction of immune cell types in the spleen are likely due to exit of these cells from the spleen and movement to the site of the tumor. This may be indicative of increased systemic circulation of these cells as previously observed in mice [39]. Macrophages, neutrophils and CD8^+^ICOS^+^ T cells were present in similar numbers between all groups (data not shown). CD8^+^ antigen specific T cells were monitored using pentamers recognizing the HY, PSA and BCG antigens (Figure 5D–F) and Figure S2. Approximately 6/7 of the combined-low dose BCG treated mice and 5/8 of the high dose BCG treated mice had BCG specific T cells. PSA specific T cells were found in 5/15 and 7/18 of the mice treated with combined-low dose BCG and high dose BCG respectively. Finally, 3/15 and 6/17 of the mice treated with combined-low dose and high dose BCG were positive for HY antigen recognizing T cells. In the combination group 2/15 (13.3%) mice had antigens to both PSA and HY while for the high dose group 4/18 (22.2%) of mice had T cells to both antigens. In the age matched control group, no mice (0/3) had antigen specific T cells. Three mice from the high dose group and one mouse in the combined-low dose group had T cells that recognized all three antigens. T cells recognizing at least one tumor antigen were found in 9/18 of the high dose group and 6/15 of the combined-low dose group (15). The pentamer assay was limited to the antigens for which pentamers were available and there may have been T cells generated against other tumor antigens.

## 4. Discussion

BCG Tokyo was more immunostimulatory than BCG Tice but not as immunostimulatory as BCG Connaught in terms of immune activation in the in vitro analyses. The results for BCG Tice were consistent with clinical reports of its reduced efficacy as compared to BCG Connaught, unless provided with maintenance therapy [19,40]. BCG Tokyo did not display direct anti-proliferative effects on MB49PSA cells but induced cell death on RAW macrophages. This may be associated with BCG internalization [41,42], which occurs rapidly in macrophages. Overall, all cytokines increased with longer exposure to BCG and with higher dose, but recombinant IFNα did not enhance this effect.

In the orthotopic murine model, low dose BCG Tokyo alone and high dose BCG when combined with *GMCSF* and *IFNα* therapy were not successful as an immunotherapeutic agent on a short therapy course of four instillations. However, high dose BCG was as effective as low dose BCG combined with *GMCSF* and *IFNα* therapy. It is possible that overstimulation, with high dose BCG and cytokine gene therapy, may have led to T cell exhaustion with PD-1 expression [43] and local inhibition at the tumor site through T regulatory cells and tissue associated macrophages [44]. This result was the opposite of what was observed with high dose BCG Tice, which was enhanced by cytokine gene therapy [31]. This is consistent with the observation in this study, that BCG Tice is not as immunostimulatory as BCG Tokyo.

Gene expression analysis post therapy revealed similar changes in cured versus tumor bearing mice in all therapy groups. Zhang et al. found that *IL24* was induced by the ras family of oncogenes in murine cells [45] and MB49 cells are known to express mutant ras [46]. Thus, the decrease in *IL24* is probably a measure of reduction of the tumor mass. Both *Krt5,* which is highly expressed in the basal layer of the urothelium and involved in urothelium regeneration [47], and *Cldn8*, which modulates barrier function [48], may reflect healing in the bladder. *Arg1* is expressed by tumor cells, myeloid derived suppressor cells and tumor associated macrophages. It converts arginine to ornithine and urea thus depleting arginine, which is required by T cells and NK cells for proliferation and is the substrate for nitric oxide production by *Nos2*. Hence, high levels of *Arg1* are immunosuppressive [49]. Generally, *Arg1*, *Nos2* and *CD163* together represent an immunosuppressive environment but BCG treatment only impacted *Arg1* expression. This is in contrast to the study by Liu et al. who found that *Arg1* expression was not modulated in RAW macrophages when stimulated with BCG Shanghai [50]. Thus, *Arg1* modulation may be a strain specific effect or an effect that is only observed in vivo. *Odc1* promotes tumor growth by increasing availability of polyamines from ornithine and acts downstream from *Arg1* [51]. Thus, depletion of both would be explained by the absence of tumor in cured mice. Similarly, *MMPs 7* [52], *10* [53] and *13* [54] are involved in bladder tumor growth and metastasis and *CCL24* is implicated in angiogenesis [55], thus reduction in expression of these genes would be consistent with tumor reduction. The array analysis did not display any differences between the tumor bearing mice of different therapy groups. This suggests that the mechanisms of anti-tumor action are similar in high dose BCG and combined–low dose BCG therapy.

Previously, we reported that mice with subcutaneously implanted bladder tumor cells, which responded to BCG Connaught immunotherapy, were resistant to subsequent subcutaneous tumor re-challenge [56]. This study shows that intravesical therapy with BCG Tokyo induced a similar systemic immunity leading to the rejection of subcutaneous tumors in cured mice but not in age matched naïve mice. This systemic immunity was associated with the presence of increased antigen specific T cells and circulating CD4^+^ICOS^+^ T cells and NK cells.

Intravesical BCG Tokyo instillations generated antigen specific T cells that recognized BCG, HY and PSA antigens. Similarly, BCG Connaught has been shown to induce BCG specific T cells after intravesical instillation [19] However, not all animals had T cells that recognized all 3 antigens. It is likely that T cells recognizing other tumor antigens were generated but were not detected as the antigens have not yet been identified. Interestingly, the mice with small tumors also resisted the subcutaneous tumors and had antigen specific T cells. Thus, the anti-tumor effects may not be solely due to T cell activity. This is consistent with the known immune cell types that have been implicated in the response to BCG immunotherapy [57,58].

This study revealed two interesting discoveries that correlate with clinical observations. First, cessation of therapy does not correlate with a cessation of on-going immune activity or surveillance. Urinary PSA monitoring showed that even after therapy cessation, tumor growth and reduction was a dynamic process, which indicates active engagement of the immune system. At day 42 post therapy, only about 33% of mice were cured but by day 84, 63% were cured in the high dose BCG group. Similar changes were observed for the combined-low dose BCG group as well. Therefore, it is reasonable to suggest that BCG treated patients receive an adequate period of follow-up before being termed BCG failures.

Secondly, BCG Tokyo induced systemic tumor antigen specific T cell responses. T cell activation may explain the greater durability of the BCG response compared to intravesical chemotherapy in some patients. Using BCG Pasteur, it has also been shown that there is induction of antigen specific T cells in mice and that it was CD4 T cells that were important [18]. Many tumor markers have been identified for bladder cancer but are variably expressed in tumors. At present, NY-ESO-1 and LAGE-1 have been identified as markers of high grade transitional cell carcinoma [59,60]. Tumor marker identification combined with immune activation studies, such as the analysis of T cell responses to BCG or tumor antigens [61], may help to individually tailor therapy and surveillance to patient need. This may reduce the cost of managing bladder cancer [62].

One question raised by this study is whether the systemic effects observed are specific to BCG Tokyo. This is an issue that needs to be addressed as many different strains of BCG are currently used in clinical therapy and the literature abounds with contradicting reports. For example, prior BCG vaccination, which is associated with the presence of BCG specific T cells [20], in one patient cohort showed a positive correlation [20] with outcomes and in another cohort showed none [16]. The strain used in the first study was BCG Connaught and in the second BCG-medac RIVM strain. However, recent murine studies show that BCG specific T cells do not reduce tumors [18]. Thus, the value of the development of BCG specific T cells is uncertain.

## 5. Conclusions

Our results indicate that a short course of high dose BCG Tokyo or low-dose BCG Tokyo combined with cytokine gene therapy induced good immune stimulation and durable tumor cures. This study provides the rationale for the design of new clinical trials to modify therapy for patients with NMIBC. Our study also suggests that tumor recurrence may not always necessitate tumor removal but that patients should be monitored to determine if their immune systems can eventually remove the tumors.

## Supplementary Materials

The following are available online at https://www.mdpi.com/article/10.3390/biomedicines9121766/s1, Figure S1: Coding RNA expression, Figure S2: Flow analysis of antigen specific T cells, Table S1: Genes selected from the Affymetrix array, Table S2: Gene expression in the bladder of mice.

## References

[1] Lamm D.L. (2000). Efficacy and safety of bacille Calmette-Guerin immunotherapy in superficial bladder cancer. Clin. Infect. Dis..

[2] Zhang W., Zhang Y., Zheng H., Pan Y., Liu H., Du P. (2013). Genome sequencing and analysis of BCG vaccine strains. PLoS ONE.

[3] Han J., Gu X., Li Y., Wu Q. (2020). Mechanisms of BCG in the treatment of bladder cancer-current understanding and the prospect. Biomed. Pharmacother..

[4] Secanella-Fandos S., Luquin M., Julian E. (2013). Connaught and Russian strains showed the highest direct antitumor effects of different Bacillus Calmette-Guerin substrains. J. Urol..

[5] Noon A.P., Kulkarni G.S. (2014). All bacillus Calmette-Guerin (BCG) strains are equal, but some BCG strains are more equal than others. Eur. Urol..

[6] Sengiku A., Ito M., Miyazaki Y., Sawazaki H., Takahashi T., Ogura K. (2013). A prospective comparative study of intravesical bacillus Calmette-Guerin therapy with the Tokyo or Connaught strain for nonmuscle invasive bladder cancer. J. Urol..

[7] Taniguchi K., Koga S., Nishikido M., Yamashita S., Sakuragi T., Kanetake H. (1999). Systemic immune response after intravesical instillation of bacille Calmette-Guerin (BCG) for superficial bladder cancer. Clin. Exp. Immunol..

[8] Kamat A.M., Briggman J., Urbauer D.L., Svatek R., Nogueras Gonzalez G.M., Anderson R. (2016). Cytokine Panel for Response to Intravesical Therapy (CyPRIT): Nomogram of Changes in Urinary Cytokine Levels Predicts Patient Response to Bacillus Calmette-Guerin. Eur. Urol..

[9] Redelman-Sidi G., Glickman M.S., Bochner B.H. (2014). The mechanism of action of BCG therapy for bladder cancer—A current perspective. Nat. Rev. Urol..

[10] Luo Y., Yamada H., Evanoff D.P., Chen X. (2006). Role of Th1-stimulating cytokines in bacillus Calmette-Guerin (BCG)-induced macrophage cytotoxicity against mouse bladder cancer MBT-2 cells. Clin. Exp. Immunol..

[11] Luo Y., Han R., Evanoff D.P., Chen X. (2010). Interleukin-10 inhibits Mycobacterium bovis bacillus Calmette-Guerin (BCG)-induced macrophage cytotoxicity against bladder cancer cells. Clin. Exp. Immunol..

[12] Joudi F.N., Smith B.J., O’Donnell M.A. (2006). Final results from a national multicenter phase II trial of combination bacillus Calmette-Guerin plus interferon alpha-2B for reducing recurrence of superficial bladder cancer. Urol. Oncol..

[13] Smith S.G., Zaharoff D.A. (2016). Future directions in bladder cancer immunotherapy: Towards adaptive immunity. Immunotherapy.

[14] Lim C.J., Nguyen P.H.D., Wasser M., Kumar P., Lee Y.H., Nasir N.J.M. (2020). Immunological Hallmarks for Clinical Response to BCG in Bladder Cancer. Front. Immunol..

[15] Kates M., Matoso A., Choi W., Baras A.S., Daniels M.J., Lombardo K. (2020). Adaptive Immune Resistance to Intravesical BCG in Non-Muscle Invasive Bladder Cancer: Implications for Prospective BCG-Unresponsive Trials. Clin. Cancer Res..

[16] Elsäßer J., Janssen M.W., Becker F., Suttmann H., Schmitt K., Sester U. (2013). Antigen-specific CD4 T cells are induced after intravesical BCG-instillation therapy in patients with bladder cancer and show similar cytokine profiles as in active tuberculosis. PLoS ONE.

[17] Kates M., Nirschl T., Sopko N.A., Matsui H., Kochel C.M., Reis L.O. (2017). Intravesical BCG Induces CD4(+) T-Cell Expansion in an Immune Competent Model of Bladder Cancer. Cancer Immunol. Res..

[18] Antonelli A.C., Binyamin A., Hohl T.M., Glickman M.S., Redelman-Sidi G. (2020). Bacterial immunotherapy for cancer induces CD4-dependent tumor-specific immunity through tumor-intrinsic interferon-γ signaling. Proc. Natl. Acad. Sci. USA.

[19] Rentsch C.A., Birkhauser F.D., Biot C., Gsponer J.R., Bisiaux A., Wetterauer C. (2014). Bacillus Calmette-Guerin Strain Differences Have an Impact on Clinical Outcome in Bladder Cancer Immunotherapy. Eur. Urol..

[20] Biot C., Rentsch C.A., Gsponer J.R., Birkhauser F.D., Jusforgues-Saklani H., Lemaitre F. (2012). Preexisting BCG-specific T cells improve intravesical immunotherapy for bladder cancer. Sci. Transl. Med..

[21] Lamm D.L., Blumenstein B.A., Crissman J.D., Montie J.E., Gottesman J.E., Lowe B.A. (2000). Maintenance bacillus Calmette-Guerin immunotherapy for recurrent TA, T1 and carcinoma in situ transitional cell carcinoma of the bladder: A randomized Southwest Oncology Group Study. J. Urol..

[22] Martínez-Piñeiro J.A., Flores N., Isorna S., Solsona E., Sebastián J.L., Pertusa C. (2002). Long-term follow-up of a randomized prospective trial comparing a standard 81 mg dose of intravesical bacille Calmette-Guérin with a reduced dose of 27 mg in superficial bladder cancer. BJU Int..

[23] Oddens J., Brausi M., Sylvester R., Bono A., van de Beek C., van Andel G. (2013). Final results of an EORTC-GU cancers group randomized study of maintenance bacillus Calmette-Guerin in intermediate- and high-risk Ta, T1 papillary carcinoma of the urinary bladder: One-third dose versus full dose and 1 year versus 3 years of maintenance. Eur. Urol..

[24] D’Andrea D., Gontero P., Shariat S.F., Soria F. (2019). Intravesical bacillus Calmette-Guérin for bladder cancer: Are all the strains equal?. Transl. Androl. Urol..

[25] Li F., Liu X., Niu H., Lv W., Han X., Zhang Y., Zhu B. (2019). Persistent stimulation with Mycobacterium tuberculosis antigen impairs the proliferation and transcriptional program of hematopoietic cells in bone marrow. Mol. Immunol..

[26] Ray D., Yung R. (2018). Immune senescence, epigenetics and autoimmunity. Clin. Immunol..

[27] Zlotta A.R., van Vooren J.P., Huygen K., Drowart A., Decock M., Pirson M., Schulman C.C. (2000). What is the optimal regimen for BCG intravesical therapy? Are six weekly instillations necessary?. Eur. Urol..

[28] De Boer E.C., Rooyakkers S.J., Schamhart D.H., de Reijke T.M., Kurth K.H. (2005). BCG dose reduction by decreasing the instillation frequency: Effects on local Th1/Th2 cytokine responses in a mouse model. Eur. Urol..

[29] Rentsch C.A., Biot C., Gsponer J.R., Bachmann A., Albert M.L., Breban R. (2013). BCG-mediated bladder cancer immunotherapy: Identifying determinants of treatment response using a calibrated mathematical model. PLoS ONE.

[30] Shah G., Zhang G., Chen F., Cao Y., Kalyanaraman B., See W.A. (2016). The Dose-Response Relationship of bacillus Calmette-Guerin and Urothelial Carcinoma Cell Biology. J. Urol..

[31] Tham S.M., Mahendran R., Chiong E., Wu Q.H., Esuvaranathan K. (2020). Gmcsf and Ifnalpha gene therapy improves the response to BCG immunotherapy in a murine model of bladder cancer. Future Oncol..

[32] Kandasamy M., Bay B.H., Lee Y.K., Mahendran R. (2011). Lactobacilli secreting a tumor antigen and IL15 activates neutrophils and dendritic cells and generates cytotoxic T lymphocytes against cancer cells. Cell Immunol..

[33] Wu Q., Esuvaranathan K., Mahendran R. (2004). Monitoring the response of orthotopic bladder tumors to granulocyte macrophage colony-stimulating factor therapy using the prostate-specific antigen gene as a reporter. Clin. Cancer Res..

[34] Tham S.M., Esuvaranathan K., Mahendran R. (2017). A Murine Orthotopic Bladder Tumor Model and Tumor Detection System. JoVE.

[35] Wu Q., Mahendran R., Esuvaranathan K. (2003). Nonviral cytokine gene therapy on an orthotopic bladder cancer model. Clin. Cancer Res..

[36] Morel C., Badell E., Abadie V., Robledo M., Setterblad N., Gluckman J.C. (2008). Mycobacterium bovis BCG-infected neutrophils and dendritic cells cooperate to induce specific T cell responses in humans and mice. Eur. J. Immunol..

[37] Esuvaranathan K., Ravuru M., Kamaraj R., Ng T.P., Chan Y.H., Cheng C.W.S., Chia S.J., Ng F.C., Feng L., Mahendran R. (2014). Long term results of double blind randomised controlled trial of interferon alpha 2b and low dose BCG in patients with high risk non-muscle invasive bladder cancer. BJU Int..

[38] Summerhayes I.C., Franks L.M. (1979). Effects of donor age on neoplastic transformation of adult mouse bladder epithelium in vitro. J. Natl. Cancer Inst..

[39] Seow S.W., Cai S., Rahmat J.N., Bay B.H., Lee Y.K., Chan Y.H. (2010). Lactobacillus rhamnosus GG induces tumor regression in mice bearing orthotopic bladder tumors. Cancer Sci..

[40] Witjes J.A., Dalbagni G., Karnes R.J., Shariat S., Joniau S., Palou J. (2016). The efficacy of BCG TICE and BCG Connaught in a cohort of 2099 patients with T1G3 non-muscle-invasive bladder cancer. Urol. Oncol..

[41] Pook S.H., Rahmat J.N., Esuvaranathan K., Mahendran R. (2007). Internalization of Mycobacterium bovis, Bacillus Calmette Guerin, by bladder cancer cells is cytotoxic. Oncol. Rep..

[42] Choi S.Y., Kim S.J., Chi B.H., Kwon J.K., Chang I.H. (2015). Modulating the internalization of bacille Calmette-Guerin by cathelicidin in bladder cancer cells. Urology.

[43] Sakuishi K., Apetoh L., Sullivan J.M., Blazar B.R., Kuchroo V.K., Anderson A.C. (2010). Targeting Tim-3 and PD-1 pathways to reverse T cell exhaustion and restore anti-tumor immunity. J. Exp. Med..

[44] Miyake M., Tatsumi Y., Gotoh D., Ohnishi S., Owari T., Iida K. (2017). Regulatory T Cells and Tumor-Associated Macrophages in the Tumor Microenvironment in Non-Muscle Invasive Bladder Cancer Treated with Intravesical Bacille Calmette-Guérin: A Long-Term Follow-Up Study of a Japanese Cohort. Int. J. Mol. Sci..

[45] Zhang R., Tan Z., Liang P. (2000). Identification of a novel ligand-receptor pair constitutively activated by ras oncogenes. J. Biol. Chem..

[46] Luo Y., Chen X., Han R., Chorev M., Dewolf W.C., O’Donnell M.A. (1999). Mutated ras p21 as a target for cancer therapy in mouse transitional cell carcinoma. J. Urol..

[47] Colopy S.A., Bjorling D.E., Mulligan W.A., Bushman W. (2014). A population of progenitor cells in the basal and intermediate layers of the murine bladder urothelium contributes to urothelial development and regeneration. Dev. Dyn..

[48] Acharya P., Beckel J., Ruiz W.G., Wang E., Rojas R., Birder L. (2004). Distribution of the tight junction proteins ZO-1, occludin, and claudin-4, -8, and -12 in bladder epithelium. Am. J. Physiol. Renal Physiol..

[49] Korrer M.J., Zhang Y., Routes J.M. (2014). Possible role of arginase-1 in concomitant tumor immunity. PLoS ONE.

[50] Liu Q., Tian Y., Zhao X., Jing H., Xie Q., Li P. (2015). NMAAP1 Expressed in BCG-Activated Macrophage Promotes M1 Macrophage Polarization. Mol. Cells.

[51] Ye Z., Zeng Z., Shen Y., Yang Q., Chen D., Chen Z. (2019). ODC1 promotes proliferation and mobility via the AKT/GSK3β/β-catenin pathway and modulation of acidotic microenvironment in human hepatocellular carcinoma. OncoTargets Ther..

[52] Bolenz C., Knauf D., John A., Erben P., Steidler A., Schneider S.W. (2018). Decreased Invasion of Urothelial Carcinoma of the Bladder by Inhibition of Matrix-Metalloproteinase 7. Bladder Cancer.

[53] Huang X., Zhu H., Gao Z., Li J., Zhuang J., Dong Y. (2018). Wnt7a activates canonical Wnt signaling, promotes bladder cancer cell invasion, and is suppressed by miR-370-3p. J. Biol. Chem..

[54] Tan M., Gong H., Wang J., Tao L., Xu D., Bao E. (2015). SENP2 regulates MMP13 expression in a bladder cancer cell line through SUMOylation of TBL1/TBLR1. Sci. Rep..

[55] Jin L., Liu W.R., Tian M.X., Jiang X.F., Wang H., Zhou P.Y. (2017). CCL24 contributes to HCC malignancy via RhoB- VEGFA-VEGFR2 angiogenesis pathway and indicates poor prognosis. Oncotarget.

[56] Gan Y.H., Zhang Y., Khoo H.E., Esuvaranathan K. (1999). Antitumour immunity of Bacillus Calmette-Guerin and interferon alpha in murine bladder cancer. Eur. J. Cancer.

[57] Joseph M., Enting D. (2019). Immune Responses in Bladder Cancer-Role of Immune Cell Populations, Prognostic Factors and Therapeutic Implications. Front. Oncol..

[58] Pettenati C., Ingersoll M.A. (2018). Mechanisms of BCG immunotherapy and its outlook for bladder cancer. Nat. Rev. Urol..

[59] Kurashige T., Noguchi Y., Saika T., Ono T., Nagata Y., Jungbluth A. (2001). Ny-ESO-1 expression and immunogenicity associated with transitional cell carcinoma: Correlation with tumor grade. Cancer Res..

[60] Sharma P., Gnjatic S., Jungbluth A.A., Williamson B., Herr H., Stockert E. (2003). Frequency of NY-ESO-1 and LAGE-1 expression in bladder cancer and evidence of a new NY-ESO-1 T-cell epitope in a patient with bladder cancer. Cancer Immun..

[61] Horn T., Grab J., Schusdziarra J., Schmid S., Maurer T., Nawroth R. (2013). Antitumor T cell responses in bladder cancer are directed against a limited set of antigens and are modulated by regulatory T cells and routine treatment approaches. Int. J. Cancer.

[62] Botteman M.F., Pashos C.L., Redaelli A., Laskin B., Hauser R. (2003). The health economics of bladder cancer: A comprehensive review of the published literature. Pharmacoeconomics.

