# Supplementary material for: Intravesical High Dose BCG Tokyo and Low Dose BCG Tokyo with GMCSF+IFN α Induce Systemic Immunity in a Murine Orthotopic Bladder Cancer Model
Source: Biomedicines. 2021 Nov 25;9(12):1766. doi: 10.3390/biomedicines9121766 (PMC8698822; doi:10.3390/biomedicines9121766)
Supplement: Supplementary file 1 [file biomedicines-09-01766-s001.zip › biomedicines-1423682-supplementary.pdf]

Table S1. Genes selected from the Affymetrix array.

Gene	Biweight average signal (log2)		Fold change (linear)	ANOVA p value	*FDR p value
	Cured	Tumor positive			
IL24	10.93	5.18	53.57	0.000622	0.259869
MMP10	10.15	4.74	42.42	0.000235	0.228890
Nos2	9.59	6.32	9.61	0.005538	0.290689
Impdh2	12.09	10.77	2.48	0.005985	0.292648
CD163	5.3	6.32	-2.02	0.003457	0.279847
Odc1	15.68	10.41	38.64	0.028136	0.354646
MMP13	11.69	4.96	106.45	0.012620	0.319801
Cldn8	5.26	7.14	-3.68	0.041561	0.378162
CCl24	7.87	5.28	6.02	0.030540	0.356582
CD276	11.92	10.06	3.63	0.004242	0.286035
Slc2a1	14.92	10.72	18.39	0.002650	0.274277
Mmp7	9.38	5.68	13.02	0.039220	0.373190
TIMP1	8.69	6.11	5.97	0.008326	0.306424
Gene	Biweight average signal (log2)		Fold change (linear)	ANOVA p value	FDR p value
	high dose BCG	Combined-low dose			
Cldn8	7.14	4.39	6.72	0.019060	0.354117
Nos2	6.32	8.11	-3.46	0.041631	0.377803
MMP13	4.96	10.55	-48.26	0.020219	0.355430
TIMP1	6.11	8.44	-5.04	0.018467	0.352319
CD276	10.06	12.13	-4.21	0.004416	0.328931
Impdh2	10.77	12.6	-3.54	0.005144	0.328931
CCl24	5.28	7.88	-6.09	0.005417	0.328931
Slc2a1	10.72	13.39	-6.37	0.031942	0.367414
Arg1	5.89	8.7	-7.01	0.009981	0.333825

Biweight average signal (log2)-Tukey's Bi-weight1 average of exon intensity of all the samples in a condition: Bi-weight average of (sample 1 exon1 intensity + sample 2 exon1 intensity +…+ sample N exon1 intensity.

*FDR adjusted p-value based on Benjamini-Hochberg Step-Up FDR-controlling Procedure

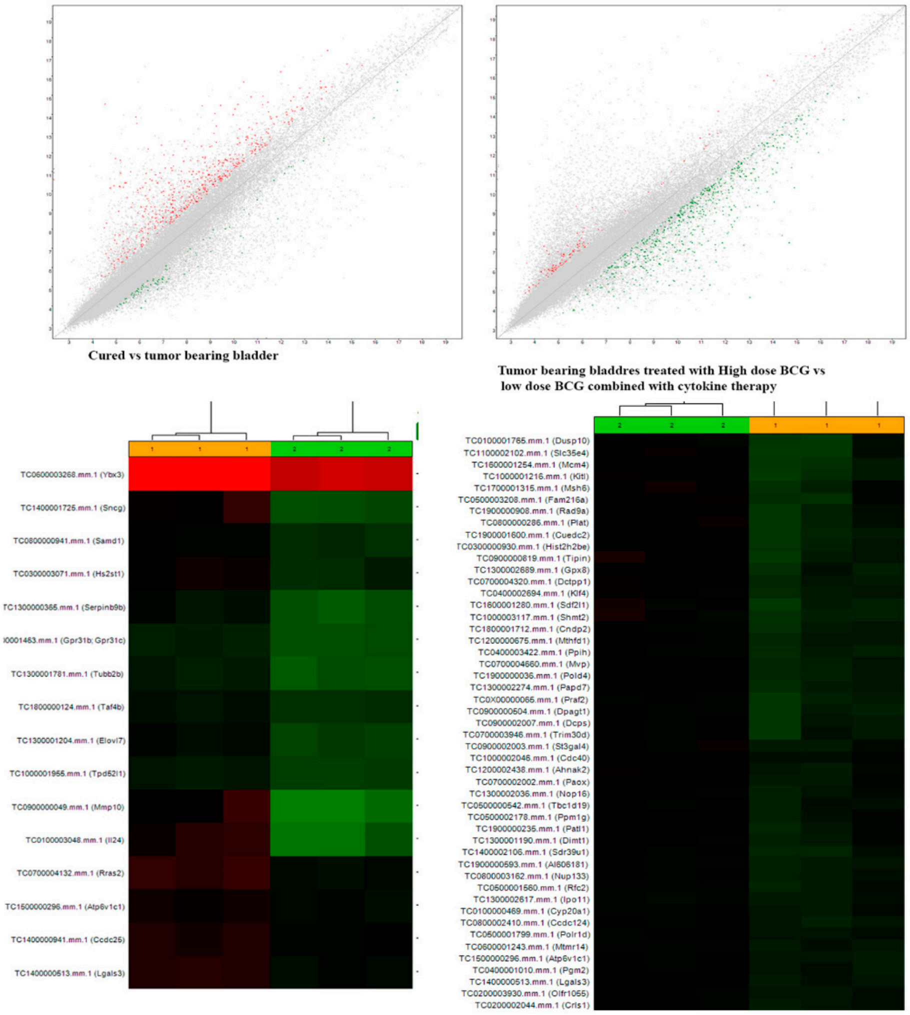

Figure S1. Coding RNA expression. A comparison of coding RNA genes between cured and tumor bearing mice indicated greater differential gene expression between cured and tumor bearing mice (left, top and bottom panel). While mice treated with high dose BCG and combined-low BCG showed fewer differentially upregulated genes and more differentially downregulated genes (right, top and bottom panel).

Table S2. Gene expression in the bladder of mice.

	pBUD + BCG(10^6)		pBUD + BCG(10^7)		GMCSF-IFN + BCG(10^6)		pBUD + Saline
	**Cured**	**Tumor**	**Cured**	**Tumor**	**Cured**	**Tumor**	**Tumor**
**N**	5	5	10	4	9	6	5
PSA(CT)	40.0 ± 0.0*	25.09 ± 3.79	38.56 ± 2.23*	25.98 ± 4.36	39.48 ± 1.57*	26.39 ± 3.75	26.23 ± 3.31
Arg1	0.053 ± 0.076	0.268 ± 0.236	0.131 ± 0.276	0.283 ± 0.290	0.090 ± 0.210*	0.390 ± 0.323	0.239 ± 0.278
Arg1/Nos2	0.140 ± 0.094	0.737 ± 0.679	0.191 ± 0.255	0.441 ± 0.327	0.182 ± 0.170	1.733 ± 1.590	0.783 ± 0.417
Ccl24	0.022 ± 0.038	1.552 ± 3.117	0.005 ± 0.008	0.196 ± 0.230	0.008 ± 0.012*	0.348 ± 0.266	0.028 ± 0.033
CD163	28.950 ± 34.761	25.472 ± 46.895	31.742 ± 41.966	10.314 ± 13.548	27.592 ± 23.518	143.249 ± 253.290	16.660 ± 21.279
CD274	0.083 ± 0.051	0.359 ± 0.309	0.477 ± 0.726	0.267 ± 0.187	0.243 ± 0.191	0.359 ± 0.183	0.466 ± 0.440
Cd276	2.019 ± 3.749	0.889 ± 1.338	1.196 ± 1.812	0.430 ± 0.312	0.346 ± 0.371	0.715 ± 0.632	0.091 ± 0.153
CD86	0.145 ± 0.070	0.416 ± 0.331	0.375 ± 0.529	0.285 ± 0.285	0.278 ± 0.158	1.179 ± 0.989	0.410 ± 0.439
Cldn8	1101.32 ± 2058.52	558.107 ±1245.278	1107.49 ± 1944.70	66.853 ± 129.444	198.644 ± 238.823	1.184 ± 1.059	10.098 ± 19.000
Il10	0.073 ± 0.077	0.543 ± 0.488	0.406 ± 0.546	0.733 ± 0.597	0.183 ± 0.242	3.413 ± 3.105	0.760 ± 0.803
Il24	0.001 ± 0.002	0.296 ± 0.180	0.001 ± 0.002	0.351 ± 0.526	0.003 ± 0.007	0.238 ± 0.248	2.062 ± 2.288^
Impdh2	2.625 ± 4.642	1.579 ± 2.763	1.218 ± 1.528	0.727 ± 0.515	0.490 ± 0.405	0.880 ± 0.746	0.981 ± 1.120
Krt5	24.538 ± 37.552	22.898 ± 46.093	16.678 ± 24.543	7.784 ± 12.394	8.452 ± 5.446*	0.341 ± 0.284	6.777 ± 8.178
Mmp10	0.010 ± 0.016	0.741 ± 0.677	0.010 ± 0.013	0.235 ± 0.313	0.015 ± 0.033	0.231 ± 0.307	0.673 ± 0.699
Mmp13	0.003 ± 0.005*	0.219 ± 0.145	0.008 ± 0.013	0.101 ± 0.123	0.011 ± 0.027*	0.294 ± 0.233	0.372 ± 0.422
Mmp7	0.215 ± 0.420	8.654 ± 14.282	0.124 ± 0.172	1.282 ± 2.377	0.425 ± 0.557	0.690 ± 1.064	0.020 ± 0.023
Mst1	0.201 ± 0.171	0.66 1 ± 0.763	1.682 ± 2.530	0.708 ± 0.533	0.271 ± 0.273	0.842 ± 0.737	0.862 ± 0.892
Nos2	0.796 ± 1.034	0.424 ± 0.253	0.678 ± 0.934	0.716 ± 0.780	0.599 ± 0.841	0.468 ± 0.492	0.634 ± 1.076
Odc1	0.089 ± 0.064*	0.677 ± 0.449	0.231 ± 0.287	0.457 ± 0.437	0.131 ± 0.0103	0.658 ± 0.560	1.256 ± 1.758
Slc2a1	0.214 ± 0.260	0.406 ± 0.283	0.136 ± 0.222	0.373 ± 0.373	0.088 ± 0.099	0.604 ± 0.603	0.383 ± 0.410
Tgfb1	1.192 ± 0.772	0.915 ± 0.204	1.803 ± 2.640	1.168 ± 0.906	1.454 ± 1.149	2.970 ± 2.706	1.268 ± 1.380
Timp1	0.011 ± 0.009*	0.310 ± 0.188	0.016 ± 0.024	0.345 ± 0.371	0.014 ± 0.013*	0.668 ± 0.589	0.301 ± 0.318

* p<0.05 against respective tumor group (independent samples T-test)

^ p<0.05 against all groups (One-way ANOVA, Bonferroni)

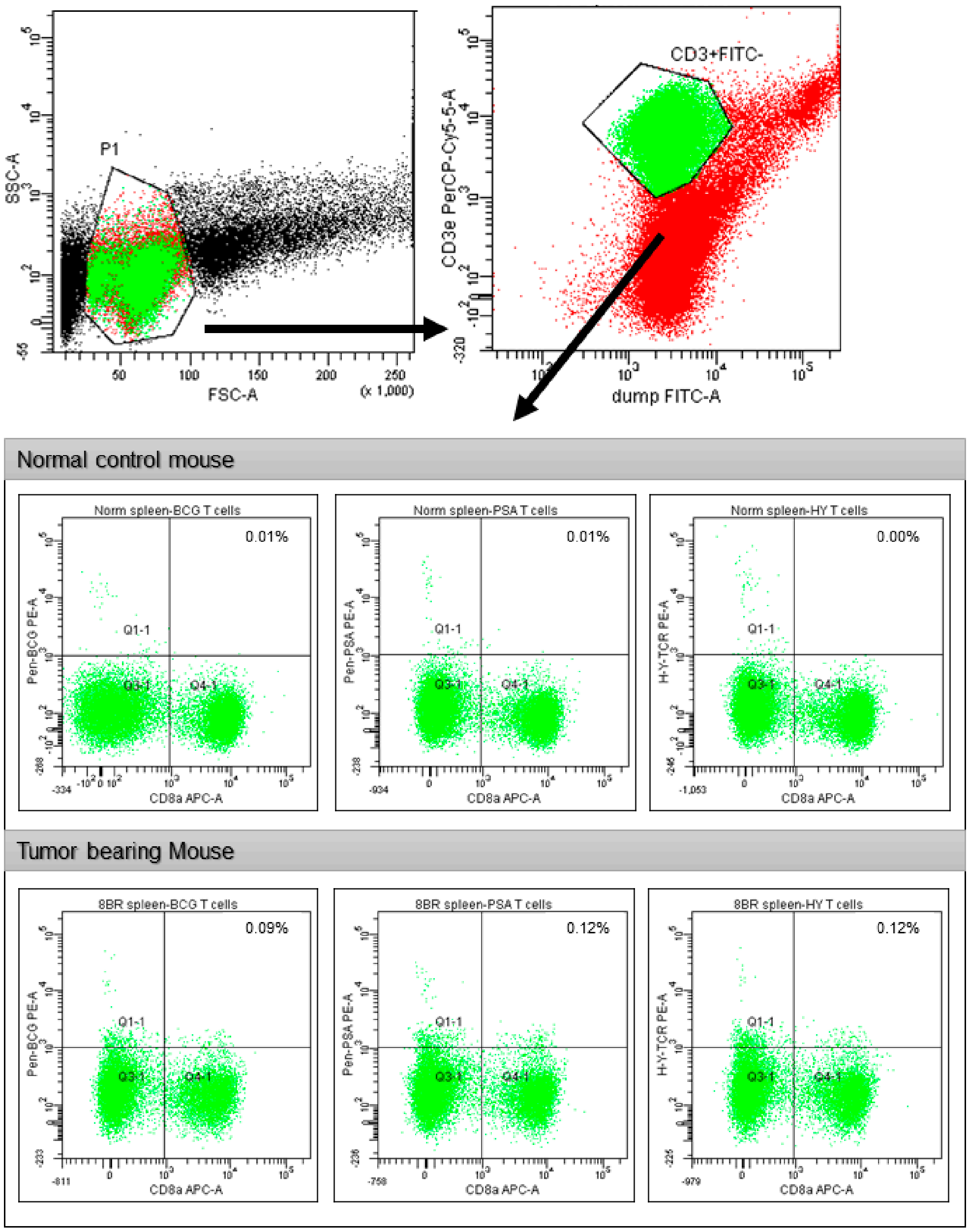

Figure S2. Flow analysis of antigen specific T cells. Lymphocytes were gated in the forward side scatter plot (top left plot). FITC labelled NK-1.1^{+}, CD11b^{+}, F4/80^{+}, CD4^{+} and CD45R/B220^{+} cells were excluded from analysis and the frequency of CD3^{+} T cells was determined. Normal spleen controls stained with the same antibodies were used to set the gate to >99% negative cells. This gate was copied to the samples stained for CD8^{+}pentamer^{+} and the frequency of positive BCG-, PSA- and HY-specific T cells were determined in control and tumor bearing mice. The percentage of antigen specific cells is shown in the quadrants.
